# South African Honey: Anti‐*Helicobacter pylori* Activity and Combined Effect With the Gut Microbiome

**DOI:** 10.1155/ijfo/9085243

**Published:** 2026-06-29

**Authors:** Sarhana Dinat, Ané Orchard, Mike Allsopp, Sandy van Vuuren

**Affiliations:** ^1^ Department of Pharmacy and Pharmacology, Faculty of Health Sciences, University of the Witwatersrand, Johannesburg, South Africa, wits.ac.za; ^2^ Honeybee Research Section, Plant Protection Research Institute, Agricultural Research Council, Stellenbosch, South Africa, arc.agric.za

**Keywords:** antimicrobial activity, gut microbiome species, hydrogen peroxide, *Lacticaseibacillus* species, *Lactobacillus* species

## Abstract

Honey has been revered for its medicinal properties for thousands of years, with medicinal uses including treating inflammation and gastric ulcers. The gut pathogen *Helicobacter pylori* is known to cause inflammation and gastric ulcers. Current treatment options for infection with *H. pylori* present several concerns due to the rapid gain in resistance and dysbiosis caused to the gut microbiome. While the anti‐*H. pylori* properties of honey have been studied globally, a comprehensive range of southern African honeys has yet to be explored against this pathogen. This study is aimed at investigating South African honey for its anti‐*H. pylori* activity and interaction with gut microbiome species. A total of 76 raw honey samples derived from South African apiarists were investigated, along with Manuka honey included for comparison. The anti‐*H. pylori* activity and interaction with the gut microbiome species were assessed using the minimum inhibitory concentration (MIC) agar dilution assay. The hydrogen peroxide (H_2_O_2_) concentration of antimicrobially active samples was determined using colorimetric test strips. A third of the investigated honeys showed activity comparable with that of Manuka honey. Honey derived from fynbos, citrus, coriander, and buchu floral sources demonstrated the best anti‐*H. pylori* activity (MIC of 6.25%). An increase in anti‐*H. pylori* activity was observed for up to 82.50% of combinations of honey and *Lactobacillus*/*Lacticaseibacillus* species. A positive correlation (R^2^ = 0.76) between increased anti‐*H. pylori* activity and increased H_2_O_2_ concentrations was observed. These results demonstrate not only the potential of South African honey as anti‐*H. pylori* agents, but also the importance of their interactions with the gut microbiome to enhance inhibition of this ulcer‐causing pathogen.

## 1. Introduction

The gut pathogen *Helicobacter pylori* has been found to colonize the majority of the global population and up to 84% of South Africans [1, 2]. Colonization by *H. pylori* causes inflammation in endothelial cells, resulting in diseases such as chronic gastritis, gastric cancer, and most notably, gastric ulcers [3]. Conventional treatment options involve the use of multiple antibiotics, a proton pump inhibitor, and bismuth [4]. These options are increasingly losing their efficacy due to *H. pylori* rapidly gaining resistance to the commonly used antibiotics, particularly in developing countries [5].

The antibiotics used for treatment have also been noted to elicit changes in the gut microbiome, impacting on groups of bacteria that are key in maintaining the hosts′ health and metabolism [6–8]. This dysbiosis in the gut microbiome has been linked to the onset of symptoms such as diarrhea, vomiting, and nausea [8]. Gut microbiome dysbiosis has also been linked to reinfection with *H. pylori*, with recurrence rates of over 50% recorded following treatment with antibiotics [9]. As such, finding alternatives that can effectively treat *H. pylori* infections with minimal impact to the gut microbiome is essential. A number of gut microbiome species such as *Lactobacillus*, *Lacticaseibacillus*, and *Bifidobacterium* species have also been noted to hold therapeutic potential. These species both occur naturally in the gut microbiome and are used supplementally to restore and maintain the gut microbiome. These species have also been found to manage gastro‐intestinal symptoms associated with *H. pylori* infections [9].

The use of honey for nutritional and medicinal purposes has been documented in numerous religious and historic texts since 5500 BC [10]. The physicochemical properties, color, composition, and flavor are determined by the floral sources used, thereby varying between geographical regions [10]. South Africa boasts a vast variety of indigenous flora. The Cape is home to one of the most diverse temperate florae globally, with the Cape Floral Kingdom recognized as a UNESCO World Heritage Site for its immense plant diversity and unique fynbos biome [11, 12]. There are over 1000 nectar‐ and pollen‐producing plant species recorded in South Africa, with at least 488 indigenous species [13, 14]. As the properties of honey are known to be dependent on the floral sources used, the honey produced from bees foraging from the diverse range of plants noted in South Africa has the potential for a multitude of unique properties.

During nectar harvesting, the enzyme glucose oxidase is added by bees to honey [15]. While this enzyme remains inactive in honey, upon dilution with water, glucose oxidase converts *β*‐D‐glucose into hydrogen peroxide (H_2_O_2_) and D‐gluconic acid [15, 16]. H_2_O_2_ has been described as one of the primary compounds responsible for the antibacterial activity of honey, with a strong correlation noted between H_2_O_2_ concentration in honey and inhibition of bacterial growth [17–19].

Honey has been shown to act as an antiulcer agent and play a role in restoring and balancing the gut microbiome [10, 20]. As *H. pylori* is causative in the onset of gastric ulcers, honey could serve as a potential treatment for *H. pylori* infections and related ailments. Several studies have assessed honey for antimicrobial activity against *H. pylori* globally [21, 22], where various types of honey from Germany, Switzerland, Iran, Oman, and Saudi Arabia were also found to possess antibacterial activity against *H. pylori* [21–23]. Manuka honey, a widely renowned honey known for medicinal purposes native to New Zealand, has been shown to have remarkable antimicrobial activity against *H. pylori*, with as little as 5% v/v completely inhibiting growth [23]. In contrast to other honey, the antimicrobial activity seen in Manuka honey is reported to be a result of the methylglyoxal present in these honey samples, with little to no H_2_O_2_ found in Manuka samples, adding to its unique properties [17, 18]. As such, Manuka honey is often regarded as the “gold standard” of medicinal honey. The investigation of southern African honey in this context has been extremely limited, despite the vast potential for unique honey from this geographical region. Only six commercial, samples of South African honey have been investigated for anti‐*H. pylori* activity [24]. Investigation of a broad range of the unique honey produced from South African floral sources has been sorely neglected. Thus, this study presents the first investigation into the anti‐*H. pylori* activity of a comprehensive range of apiarist‐derived South African honey samples. Furthermore, the anti‐*H. pylori* activity of the novel combination of South African honey and gut microbiome species was investigated, along with the first assessment of the H_2_O_2_ concentration of the antimicrobially active South African honey samples.

## 2. Materials and Methods

### 2.1. Honey Samples

**Figure 1:**
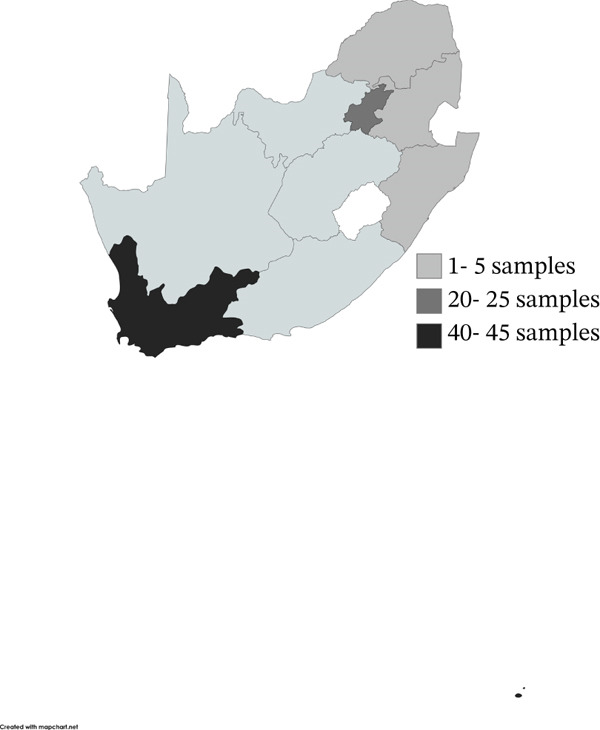
Geographic distribution of honey samples collected for this study (https://www.mapchart.net/).

A total of 76 unheated, non‐irradiated, raw honey samples were collected from various regions around South Africa from apiarists from the South African Bee Industry Organization (SABIO) (Table S1, Figure 1). A Manuka honey sample was obtained from New Zealand to serve as the “gold standard” control (Forest and Bees Native Honey, Stratford; methylglyoxal (MGO) 67+). A sample from Mozambique and three samples from Malawi were also included for further comparison. Perceived nectar sources were recorded as indicated by the beekeepers. The samples were stored away from light at ambient temperature (±20°C).

### 2.2. Bacterial Strains and Culture Conditions

Three clinical isolates of *H. pylori* were isolated from patients with gastritis (Chris Hani Baragwanath Academic Hospital; Dr. Ayodeji Idowu—African Institute of Digestive Diseases) (Ethical Clearance Number: M210891, Human Research Ethics Committee, University of the Witwatersrand). Media used for culturing consisted of brain–heart infusion (BHI) broth or Columbia blood agar (CBA) (Thermo Scientific), supplemented with *Campylobacter* selective supplements (Skirrows—Oxoid) and 7% fetal bovine serum. The *H. pylori* strains were cultured at 37°C in a micro‐aerobic environment using a micro‐aerobic gas pack (CampyGen) for 24 h in broth and 72 h on agar.

The gut microbiome species used included *Lactobacillus*/*Lacticaseibacillus* species, as these species are known to occur naturally in the gut and are used most frequently for therapeutic restoration of the gut flora. These included; *Lactobacillus acidophilus* ATCC 314, *Lacticaseibacillus casei* (formerly known as *Lactobacillus casei*) ATCC 334, and *Lacticaseibacillus rhamnosus* (formerly known as *Lactobacillus rhamnosus*) ATCC 7469 (Davies Diagnostics). All *Lactobacillus*/*Lacticaseibacillus* species were cultured in MRS broth or on MRS agar (Thermo Scientific) at 5% CO_2_ and 37°C for 24 h. Cultures were enumerated using the serial dilution method, where the colony forming units (CFU)/mL were determined for the respective cultures [25].

### 2.3. Antimicrobial Assays

Antimicrobial testing was carried out using an agar dilution assay to determine the minimum inhibitory concentration (MIC) of honey against *H. pylori* strains and against the *Lactobacillus*/*Lacticaseibacillus* species. Honey was added aseptically to molten CBA or MRS agar, in a series of concentrations (50%, 25%, 12.50%, 6.25%, and 3.13% v/v). The honey‐agar solutions were dispensed into the wells of 24‐well microtiter plates and allowed to solidify. The honey‐agar plates were inoculated with bacterial cultures (0.50 MacFarland turbidity standard) and incubated according to each species′ respective culture conditions (Section 2.2) [26]. A negative control was included, consisting of agar alone. Amoxicillin was selected as a positive control, as it is recommended most frequently for the treatment of *H. pylori* infections [4]. Amoxicillin (Sigma‐Aldrich) was included at a range of concentrations (40.00, 20.00, 10.00, 5.00, and 2.50 *μ*g/mL). The MIC values for each honey sample were taken as the lowest concentration of honey to inhibit visible growth on the agar plates. All assays were carried out in triplicate, with results reported as mean ± standard deviation. The antimicrobial activity of each honey sample was compared with that of the “gold standard,” Manuka honey, and noteworthy activity determined to be MIC values less than or equal to the MIC of Manuka honey.

The inhibition of *H. pylori* by *Lactobacillus*/*Lacticaseibacillus* species was assessed using an agar dilution assay. Cultures of each *Lactobacillus*/*Lacticaseibacillus* species were added to CBA (a volume of 1 mL of culture was added per 10 mL of agar). Each *Lactobacillus/Lacticaseibacillus* species was added to agar in a range of concentrations (0.02–3.00 × 10^8^ CFU/mL). The culture‐agar solutions were dispensed into the wells of 24‐well microtiter plates and allowed to solidify. The plates were inoculated with *H. pylori* and allowed to incubate in a micro‐aerobic environment to allow *H. pylori* to grow. The plates were observed for visible growth of *H. pylori* after 72 h, and the MIC taken as that concentration of *Lactobacillus*/*Lacticaseibacillus* that inhibited visible growth completely. Negative controls of agar alone and *Lactobacillus*/*Lacticaseibacillus* alone, and a positive control of 0.01 mg/mL of amoxicillin, were included, and the assay carried out in triplicate.

Antibiotic susceptibility of each *H. pylori* strain was determined using MIC Test Strips (Liofilchem) for amoxicillin (92021), ciprofloxacin (92045), clarithromycin (92048), metronidazole (92087), and tetracycline (92114). A volume of 100 *μ*L of *H. pylori* culture was spread onto CBA. Each MIC test strip was placed aseptically onto the plate and incubated for 72 h under micro‐aerobic conditions. The MIC was taken as the lowest concentration to inhibit visible growth. The assay was performed in triplicate, with MIC values reported as mean ± standard deviation.

### 2.4. Anti‐*H. pylori* Assay of Honey–*Lactobacillus*/*Lacticaseibacillus* Combinations

The effects of honey combined with *Lactobacillus*/*Lacticaseibacillus* species were investigated against *H. pylori* using an agar dilution assay with selective CBA. Each honey sample was added in a range of concentrations to molten CBA (Section 2.3). This was followed by aliquots of each *Lactobacillus*/*Lacticaseibacillus* species (1 mL of culture at a concentration 1 × 10^8^ CFU/mL per 10 mL of agar). The Lactobacillus/Lacticaseibacillus–honey‐agar solutions were dispensed into the wells of 24‐well microtiter plates and allowed to solidify. Aliquots of *H. pylori* culture were spread onto these agar plates and incubated at 37°C for 72 h in a micro‐aerobic environment to allow for the growth of *H. pylori* alone. The MIC was taken as the lowest concentration of honey in that combination to completely inhibit visible growth. Negative controls consisting of agar alone and of the respective *Lactobacillus*/*Lacticaseibacillus* species alone, a culture control of *H. pylori*, and a positive control of 0.01 mg/mL amoxicillin were included. All assays were performed in triplicate, and the mean reported with standard deviations summarized as a range.

### 2.5. Assessment of H_2_O_2_ Content

The concentrations of H_2_O_2_ were determined in samples that had demonstrated the best antimicrobial activity, along with the Manuka honey. Samples that demonstrated the poorest antimicrobial activity were also selected for comparison. The concentrations of H_2_O_2_ were quantified using colorimetric test strips (MQuant, Merck 110011). Honey samples were weighed and dissolved in sterile water (1 g of honey in 4 mL of water). After 1 h, the H_2_O_2_ concentrations were determined according to the manufacturers′ guide and the final H_2_O_2_ concentration reported in mg/mL/h at ~25°C [27]. The concentrations of H_2_O_2_ recorded were plotted against the antimicrobial activity of the honey samples against *H. pylori*, and the coefficient of determination (R^2^) was calculated to determine the strength of the correlation.

### 2.6. Statistical Analysis

The independent sample t‐test, performed using Microsoft Excel, was used to assess the significance of the change in MIC values of honey alone compared with honey combined with *Lactobacillus*/*Lacticaseibacillus* species, where *p* values < 0.05 were considered significant.

## 3. Results and Discussion

### 3.1. Antimicrobial Activity

The MIC values of 76 South African honey samples were determined against three clinical *H. pylori* strains (Table 1). Antimicrobial activity of each honey sample was compared with that of Manuka honey, where noteworthy activity was taken as MIC values equal to or less than the MIC of Manuka honey for that strain. The negative controls showed complete growth, whereas the positive control, amoxicillin, had an MIC of 10.00 *μ*g/mL for all three strains. A total of 24 honey samples showed noteworthy activity against at least one *H. pylori* strain, with nine samples showing noteworthy activity against all three strains. Samples 35, 43, and 59 held the best anti‐*H. pylori* activity, with mean MIC values of 6.25% v/v.

**Table 1:** The minimum inhibitory concentrations in % v/v of southern African honey samples against three clinical *H. pylori* strains and the mean across all three strains, represented at mean ± standard deviation.

Honey sample	MIC (% v/v)		
	Strain 1	Strain 2	Strain 3
1	12.50 ± 0.00	20.83 ± 7.22	12.50 ± 0.00
2	12.50 ± 0.00	**12.50** ^**a**^ ± 0.00	12.50 ± 0.00
3	**6.25** ± 0.00	**10.42** ± 3.61	**6.25** ± 0.00
4	10.42 ± 3.61	20.83 ± 7.22	16.67 ± 7.22
5	12.50 ± 0.00	**12.50** ± 0.00	12.50 ± 0.00
6	12.50 ± 0.00	16.67 ± 7.22	12.50 ± 0.00
7	8.33 ± 3.61	16.67 ± 7.22	10.42 ± 3.61
8	12.50 ± 0.00	16.67 ± 7.22	10.42 ± 3.61
9	12.50 ± 0.00	**12.50** ± 0.00	12.50 ± 0.00
10	16.67 ± 7.22	25.00 ± 0.00	12.50 ± 0.00
11	25.00 ± 0.00	41.67 ± 14.43	25.00 ± 0.00
12	12.50 ± 0.00	**12.50** ± 0.00	12.50 ± 0.00
13	**6.25** ± 0.00	16.67 ± 7.22	10.42 ± 3.61
14	25.00 ± 0.00	25.00 ± 0.00	25.00 ± 0.00
15	50.00 ± 0.00	50.00 ± 0.00	50.00 ± 0.00
16	50.00 ± 0.00	50.00 ± 0.00	50.00 ± 0.00
17	25.00 ± 0.00	25.00 ± 0.00	25.00 ± 0.00
18	12.50 ± 0.00	20.83 ± 7.22	12.50 ± 0.00
19	25.00 ± 0.00	25.00 ± 0.00	25.00 ± 0.00
20	25.00 ± 0.00	25.00 ± 0.00	25.00 ± 0.00
21	**6.25** ± 0.00	**12.50** ± 0.00	**6.25** ± 0.00
22	50.00 ± 0.00	> 50.00 ± 0.00	50.00 ± 0.00
23	33.33 ± 14.43	50.00 ± 0.00	50.00 ± 0.00
24	25.00 ± 0.00	25.00 ± 0.00	25.00 ± 0.00
25	16.67 ± 7.22	16.67 ± 7.22	10.42 ± 3.61
26	**6.25** ± 0.00	**12.50** ± 0.00	**6.25** ± 0.00
27	12.50 ± 0.00	25.00 ± 0.00	12.50 ± 0.00
28	**6.25** ± 0.00	**10.42** ± 3.61	**6.25** ± 0.00
29	> 50.00 ± 0.00	> 50.00 ± 0.00	> 50.00 ± 0.00
30	12.50 ± 0.00	**12.50** ± 0.00	12.50 ± 0.00
31	33.33 ± 14.43	33.33 ± 14.43	25.00 ± 0.00
32	33.33 ± 14.43	25.00 ± 0.00	33.33 ± 14.43
33	16.67 ± 7.22	25.00 ± 0.00	12.50 ± 0.00
34	> 50.00 ± 0.00	> 50.0 ± 0.000	> 50.00 ± 0.00
35	**6.25** ± 0.00	**6.25** ± 0.00	**6.25** ± 0.00
36	50.00 ± 0.00	50.00 ± 0.00	50.00 ± 0.00
37	50.00 ± 0.00	50.00 ± 0.00	50.00 ± 0.00
38	12.50 ± 0.00	**12.50** ± 0.00	12.50 ± 0.00
39	41.67 ± 14.43	25.00 ± 0.00	25.00 ± 0.00
40	25.00 ± 0.00	25.00 ± 0.00	25.00 ± 0.00
41	25.00 ± 0.00	25.00 ± 0.00	12.50 ± 0.00
42	25.00 ± 0.00	25.00 ± 0.00	25.00 ± 0.00
43	**6.25** ± 0.00	**6.25** ± 0.00	**6.25** ± 0.00
44	50.00 ± 0.00	50.00 ± 0.00	50.00 ± 0.00
45	12.50 ± 0.00	25.00 ± 0.00	12.50 ± 0.00
46	12.50 ± 0.00	**12.50** ± 0.00	12.50 ± 0.00
47	12.50 ± 0.00	**12.50** ± 0.00	12.50 ± 0.00
48	12.50 ± 0.00	25.00 ± 0.00	12.50 ± 0.00
49	12.50 ± 0.00	16.67 ± 7.22	12.50 ± 0.00
50	12.50 ± 0.00	25.00 ± 0.00	12.50 ± 0.00
51	12.50 ± 0.00	**12.50** ± 0.00	12.50 ± 0.00
52	> 50.00 ± 0.00	> 50.00 ± 0.00	> 50.00 ± 0.00
53	12.50 ± 0.00	**12.50** ± 0.00	12.50 ± 0.00
54	> 50.00 ± 0.00	> 50.00 ± 0.00	> 50.00 ± 0.00
55	12.50 ± 0.00	**12.50** ± 0.00	12.50 ± 0.00
56	12.50 ± 0.00	16.67 ± 7.22	12.50 ± 0.00
57	41.67 ± 14.43	50.00 ± 0.00	50.00 ± 0.00
58	25.00 ± 0.00	25.00 ± 0.00	25.00 ± 0.00
59	**6.25** ± 0.00	**6.25** ± 0.00	**6.25** ± 0.00
60	50.00 ± 0.00	50.00 ± 0.00	50.00 ± 0.00
61	**6.25** ± 0.00	**10.42** ± 3.61	**6.25** ± 0.00
62	25.00 ± 0.00	25.00 ± 0.00	25.00 ± 0.00
63	**6.25** ± 0.00	**12.50** ± 0.00	12.50 ± 0.00
64	**6.25** ± 0.00	**8.33** ± 3.61	8.33 ± 3.61
65	50.00 ± 0.00	50.00 ± 0.00	50.00 ± 0.00
66	20.83 ± 7.22	25.00 ± 0.00	20.83 ± 7.22
67	> 50.00 ± 0.00	> 50.00 ± 0.00	> 50.00 ± 0.00
68	25.00 ± 0.00	25.00 ± 0.00	25.00 ± 0.00
69	25.00 ± 0.00	41.67 ± 14.43	25.00 ± 0.00
70	**6.25** ± 0.00	**10.42** ± 3.61	**6.25** ± 0.00
71	12.50 ± 0.00	25.00 ± 0.00	12.50 ± 0.00
72	33.33 ± 14.43	50.00 ± 0.00	25.00 ± 0.00
73	12.50 ± 0.00	**12.50** ± 0.00	12.50 ± 0.00
74	25.00 ± 0.00	41.67 ± 14.43	25.00 ± 0.00
75	50.00 ± 0.00	50.00 ± 0.00	50.00 ± 0.00
76	12.50 ± 0.00	16.67 ± 7.22	12.50 ± 0.00
A1	25.00 ± 0.00	50.00 ± 0.00	25.00 ± 0.00
A2	50.00 ±0.00	50.00 ± 0.00	50.00 ± 0.00
A3	50.00 ± 0.00	50.00 ± 0.00	50.00 ± 0.00
A4	33.33 ± 14.43	50.00 ± 0.00	50.00 ± 0.00
M	6.25 ± 0.00	12.50 ± 0.00	6.25 ± 0.00

^a^Noteworthy antimicrobial activity (≤ MIC of Manuka honey for each strain) denoted in bold.

Numerous samples of South African honey that demonstrated noteworthy activity were of Western Cape origin. The Cape is noted to house one of the most diverse temperate florae in the world, with over 3000 plants recorded for therapeutic use [11, 28]. Perceived floral sources of honey with noteworthy activity included African basil, coriander, citrus, plum, various fynbos such as coastal and mountain fynbos, and buchu. Although African basil‐ or coriander‐derived honey samples were not previously investigated against *H. pylori*, plant extracts of African basil (*Ocimum suave* L.) and coriander (*Eryngium foetidum* L.) have previously shown antimicrobial activity against *H. pylori* [29, 30].

Citrus‐derived honey had an average MIC of 6.25% v/v in this study. These findings are consistent with those previously reported, where citrus‐derived honey was found to be one of the most active honeys when investigated against *H. pylori.* These honey samples demonstrated MIC values ranging from 0.01% to 10.00% v/v and were found to inhibit the enzyme urease, an enzyme used by *H. pylori* during growth and when colonizing the gastric mucosa [23, 31, 32]. Inhibition of urease could thus be a potential mechanism of the antimicrobial activity of honey noted against *H. pylori.*

Fynbos‐derived honey was found to inhibit clinical *H. pylori* strains in this study at various concentrations (6.25%–50.00% v/v), which aligns with the anti‐*H. pylori* activity previously reported, where fynbos‐derived honey demonstrated antimicrobial activity against clinical *H. pylori* strains at a range of concentrations (10.00%–50.00% v/v) [23]. Fynbos‐derived honey also showed varying antimicrobial activity against various *Streptococcus* species as well as against common bacterial wound pathogens [26, 33]. The wide variation of activity observed in fynbos‐derived honey could be the result of differences in geographic location, or variable species of fynbos used as the floral sources. Although buchu‐derived honey has not previously been studied against *H. pylori*, buchu (*Agathosma betulina* [P.J. Bergius] Pillans and *Agathosma crenulata* [L.] Pillans) has been used traditionally in South Africa for treating gastric ulcers and has been shown to have anti‐inflammatory activity [34, 35].

The honey samples investigated from other African countries in this study showed moderate anti‐*H. pylori* activity. A mean MIC of 44.44% was recorded for the Mozambican macadamia‐derived honey (Sample A4), whereas the South African Gauteng macadamia‐derived honey (Sample 19) had a mean MIC of 25.00%. This could be attributed to the difference in biomes and climates between the regions from which the samples were harvested, as Mozambique is predominantly made up of savannah biomes and has a warmer, wetter climate, whereas Gauteng is predominantly made up of grassland, with a comparatively cooler, drier climate [36, 37]. Interestingly, the South African Mpumalanga macadamia‐derived honey (Sample 29) showed similar activity (> 50.00% v/v) to that of the Mozambique sample. This could be due to the proximity of the regions of origin, as the area of origin of Sample 29, Barberton, is geographically closer to Mozambique than Gauteng. African honey has been poorly explored for its antimicrobial activity against *H. pylori* [38]. In a study investigating several Nigerian honey variants, *H. pylori* were found to be the most susceptible [39]. The results from this study as well as that of Ademokoya [38] show the potential of African honey to be utilized in treating *H. pylori* infections.

When investigating any inhibitory effect the honey samples might hold against the *Lactobacillus*/*Lacticaseibacillus* species, no inhibition at the highest concentration tested was observed for all three species. This favorable outcome indicates that the investigated honey samples do not hinder the growth of naturally occurring gut microbiome species that play a key role in maintaining gut health. This is in contrast with conventionally used antibiotics that have been shown to result in gut microbiome dysbiosis.

When investigating the inhibition of *Lactobacillus*/*Lacticaseibacillus* species against *H. pylori*, a mean MIC value of 1.50 × 10^8^ CFU/mL was recorded for *L. acidophilus*, *L. casei*, and *L. rhamnosus*. This indicates that the *Lactobacillus*/*Lacticaseibacillus* species exhibit inhibitory effects on the *H. pylori* strains. *Lactobacillus*/*Lacticaseibacillus* species have been noted to employ several mechanisms of action when inhibiting *H. pylori* infections, such as decreasing inflammatory cytokines and interfering with adhesion, thereby reducing its ability to colonize the gastric mucosa [40].

The antibiotic susceptibility profile of each of the clinical *H. pylori* strains was determined and is summarized in Table 2. The antibiotics assessed included those most commonly recommended for treatment of *H. pylori* infections [4]. The results indicated that all three strains were most susceptible to amoxicillin and showed complete resistance to metronidazole. The overall trend indicated variability in the susceptibility of each of the strains, with Strain 1 being the most susceptible to amoxicillin, clarithromycin, and ciprofloxacin. Clinical strains have typically been shown to have varying antimicrobial susceptibility [41]. This variability is often seen in isolated strains, as each strain is noted to hold unique resistance mechanisms and mutations, as a result of the unique antimicrobial and environmental exposures, which can also lead to hetero‐resistance within strains isolated from a single host [29].

**Table 2:** The minimum inhibitory concentrations in *μ*g/mL of antibiotics against three clinical *H. pylori* strains, represented at mean ± standard deviation.

Antibiotic	MIC (*μ*g/mL)		
	Strain 1	Strain 2	Strain 3
Amoxicillin	0.13 ± 0.00	0.13 ± 0.00	0.19 ± 0.00
Clarithromycin	0.38 ± 0.00	0.50 ± 0.00	1.00 ± 0.00
Metronidazole	Complete resistance	Complete resistance	Complete resistance
Tetracycline	0.75 ± 0.00	0.75 ± 0.00	0.75 ± 0.00
Ciprofloxacin	0.16 ± 0.00	0.31 ± 0.00	0.31 ± 0.00

### 3.2. Anti‐*H. pylori* Assay of Honey–*Lactobacillus*/*Lacticaseibacillus* Combinations

The interaction of gut microbiome species with honey was investigated for its antimicrobial activity against *H. pylori* (Table 3). The overall trend showed a significant increase in the anti‐*H. pylori* activity when *Lactobacillus*/*Lacticaseibacillus* species were combined with honey, compared with that of the honey alone when exposed to *H. pylori*. Complete inhibition was observed for the positive control. No growth was observed for the negative controls, where selective CBA was inoculated with each *Lactobacillus*/*Lacticaseibacillus* species. The culture controls, where *H. pylori* alone were inoculated onto selective CBA, showed growth. These results indicated that the selective CBA allowed for the growth of *H. pylori* only, and not the *Lactobacillus*/*Lacticaseibacillus* species, when results of the combination assays were recorded.

**Table 3:** The minimum inhibitory concentrations in % v/v of honey combined with one of three *Lactobacillus*/*Lacticaseibacillus* species against three *H. pylori* strains.

Honey sample	MIC (% v/v)								
	*L. acidophilus*			*L. casei*			*L. rhamnosus*		
	Strain 1	Strain 2	Strain 3	Strain 1	Strain 2	Strain 3	Strain 1	Strain 2	Strain 3
1	12.50	**12.50** ^**a**^	12.50	12.50	**12.50**	12.50	12.50	**12.50**	12.50
2	**6.25**	12.50	**6.25**	**6.25**	**6.25**	**6.25**	**6.25**	12.50	**6.25**
3	6.25	**6.25**	6.25	**3.13**	**6.25**	**3.13**	**3.13**	**6.25**	**3.13**
4	20.83	**12.50**	25.00	**6.25**	**8.33**	**6.25**	**6.25**	**12.50**	**6.25**
5	25.00	12.50	25.00	12.50	12.50	12.50	**6.25**	12.50	**6.25**
6	**6.25**	**6.25**	**6.25**	**6.25**	**6.25**	**6.25**	**3.13**	**6.25**	**3.13**
7	**6.25**	**6.25**	**6.25**	**6.25**	**6.25**	**6.25**	**6.25**	**6.25**	**6.25**
8	**6.25**	**6.25**	**6.25**	**3.13**	**3.13**	**3.13**	**3.13**	**3.13**	**3.13**
9	12.50	**6.25**	**10.42**	**6.25**	**6.25**	**6.25**	**6.25**	**6.25**	**6.25**
10	**12.50**	**12.50**	12.50	**6.25**	**12.50**	**6.25**	**6.25**	**6.25**	**6.25**
11	25.00	**12.50**	**20.83**	**6.25**	**8.33**	**6.25**	**6.25**	**12.50**	**6.25**
12	**6.25**	**6.25**	**6.25**	**3.13**	**6.25**	**3.13**	**3.13**	**6.25**	**3.13**
13	12.50	**12.50**	12.50	**3.13**	**5.21**	**3.13**	**3.13**	**6.25**	**3.13**
14	**12.50**	**12.50**	**12.50**	**6.25**	**6.25**	**6.25**	**6.25**	**12.50**	**6.25**
15	**25.00**	**12.50**	**25.00**	**6.25**	**12.50**	**6.25**	**6.25**	**6.25**	**6.25**
16	**25.00**	**25.00**	**25.00**	**12.50**	**20.83**	**12.50**	**12.50**	**12.50**	**12.50**
17	**6.25**	**6.25**	**6.25**	**3.13**	**6.25**	**3.13**	**3.13**	**6.25**	**3.13**
18	25.00	**12.50**	25.00	12.50	**12.50**	12.50	**6.25**	**12.50**	**6.25**
19	25.00	25.00	25.00	**12.50**	**20.83**	**12.50**	**12.50**	**12.50**	**12.50**
20	**12.50**	25.00	**12.50**	**3.13**	**6.25**	**3.13**	**3.13**	**6.25**	**3.13**
21	6.25	**6.25**	6.25	6.25	**6.25**	6.25	6.25	**6.25**	6.25
22	**12.50**	**12.50**	**12.50**	**25.00**	**20.83**	**25.00**	**25.00**	**25.00**	**25.00**
23	**6.25**	**6.25**	**6.25**	**12.50**	**8.33**	**12.50**	**3.13**	**6.25**	**3.13**
24	**6.25**	**6.25**	**6.25**	**6.25**	**6.25**	**6.25**	**6.25**	**6.25**	**6.25**
25	**12.50**	**12.50**	12.50	**12.50**	**12.50**	12.50	**12.50**	**12.50**	12.50
26	6.25	12.50	6.25	6.25	12.50	6.25	6.25	12.50	6.25
27	12.50	**6.25**	12.50	**6.25**	**6.25**	**6.25**	**6.25**	**6.25**	**6.25**
28	6.25	**12.50**	6.25	6.25	**8.33**	6.25	12.50	12.50	12.50
29	50.00	50.00	50.00	50.00	50.00	50.00	50.00	50.00	50.00
30	12.50	12.50	12.50	25.00	12.50	25.00	25.00	25.00	25.00
31	**25.00**	**25.00**	25.00	**6.25**	**12.50**	**6.25**	**12.50**	**25.00**	**12.50**
32	**12.50**	**12.50**	**12.50**	**6.25**	**12.50**	**6.25**	**12.50**	**12.50**	**12.50**
33	**6.25**	**6.25**	**6.25**	**3.13**	**6.25**	**3.13**	**3.13**	**6.25**	**3.13**
34	**6.25**	**12.50**	**8.33**	**12.50**	**12.50**	**12.50**	**12.50**	**12.50**	**12.50**
35	6.25	6.25	6.25	6.25	6.25	6.25	6.25	6.25	6.25
36	**25.00**	**12.50**	**16.67**	**25.00**	**16.67**	**16.67**	**12.50**	**12.50**	**12.50**
37	50.00	50.00	50.00	**25.00**	**41.67**	**25.00**	**25.00**	50.00	**25.00**
38	12.50	12.50	12.50	12.50	12.50	12.50	12.50	12.50	12.50
39	**6.25**	**12.50**	**8.33**	**6.25**	**12.50**	**6.25**	**6.25**	**6.25**	**6.25**
40	**12.50**	**12.50**	**12.50**	**6.25**	**12.50**	**6.25**	**6.25**	**12.50**	**10.42**
41	25.00	25.00	25.00	**6.25**	**12.50**	**6.25**	**16.67**	25.00	12.50
42	**6.25**	**6.25**	**6.25**	**3.13**	**6.25**	**3.13**	**3.13**	**6.25**	**3.13**
43	6.25	6.25	6.25	6.25	6.25	6.25	6.25	6.25	6.25
44	**12.50**	**12.50**	**12.50**	**25.00**	**12.50**	**20.83**	**6.25**	**12.50**	**6.25**
45	12.50	**12.50**	12.50	12.50	**12.50**	12.50	12.50	**12.50**	12.50
46	**6.25**	**6.25**	**6.25**	**6.25**	**6.25**	**6.25**	**3.13**	**6.25**	**3.13**
47	12.50	12.50	12.50	12.50	12.50	12.50	12.50	12.50	12.50
48	12.50	**12.50**	12.50	**6.25**	**8.33**	**6.25**	12.50	**12.50**	12.50
49	12.50	**12.50**	12.50	12.50	**12.50**	12.50	12.50	**12.50**	12.50
50	12.50	**12.50**	12.50	**6.25**	**12.50**	**6.25**	**6.25**	**8.33**	**6.25**
51	12.50	12.50	12.50	**6.25**	**10.42**	**6.25**	**6.25**	**6.25**	**6.25**
52	**12.50**	**12.50**	**12.50**	**12.50**	**12.50**	**12.50**	**12.50**	**12.50**	**12.50**
53	12.50	12.50	12.50	12.50	12.50	12.50	12.50	12.50	12.50
54	**25.00**	**12.50**	**20.83**	**25.00**	**16.67**	**20.83**	**12.50**	**12.50**	**12.50**
55	12.50	12.50	12.50	12.50	12.50	12.50	12.50	12.50	12.50
56	12.50	**12.50**	12.50	12.50	**12.50**	12.50	12.50	**12.50**	12.50
57	**6.25**	**12.50**	**6.25**	**3.13**	**5.21**	**3.13**	**3.13**	**6.25**	**3.13**
58	25.00	25.00	25.00	25.00	25.00	25.00	25.00	25.00	25.00
59	6.25	6.25	6.25	6.25	6.25	6.25	6.25	6.25	6.25
60	**25.00**	**25.00**	**25.00**	**25.00**	**25.00**	**25.00**	**25.00**	**25.00**	**25.00**
61	6.25	**6.25**	6.25	6.25	**6.25**	6.25	6.25	**6.25**	6.25
62	25.00	**12.50**	**16.67**	**12.50**	**12.50**	**12.50**	**12.50**	**12.50**	**12.50**
63	6.25	12.50	**6.25**	12.50	12.50	12.50	12.50	12.50	12.50
64	6.25	**6.25**	**6.25**	6.25	**6.25**	**6.25**	6.25	**6.25**	**6.25**
65	**12.50**	**12.50**	**8.33**	**25.00**	**20.83**	**25.00**	**12.50**	**12.50**	**25.00**
66	**25.00**	25.00	**25.00**	**25.00**	25.00	**25.00**	**25.00**	25.00	**25.00**
67	**12.50**	**25.00**	**12.50**	**12.50**	**25.00**	**12.50**	**12.50**	**25.00**	**12.50**
68	**12.50**	**12.50**	**12.50**	**12.50**	**12.50**	**8.33**	**6.25**	**12.50**	**6.25**
69	25.00	**25.00**	25.00	25.00	**25.00**	25.00	25.00	**25.00**	25.00
70	6.25	**6.25**	6.25	6.25	**6.25**	6.25	6.25	**6.25**	6.25
71	12.50	**12.50**	12.50	**6.25**	**12.50**	**6.25**	**6.25**	**6.25**	**6.25**
72	**12.50**	**25.00**	**16.67**	**12.50**	**12.50**	**12.50**	**12.50**	**20.83**	**12.50**
73	12.50	12.50	12.50	12.50	12.50	12.50	12.50	12.50	12.50
74	**6.25**	**12.50**	**6.25**	**6.25**	**12.50**	**6.25**	**6.25**	**6.25**	**6.25**
75	**6.25**	**10.42**	**6.25**	**3.13**	**6.25**	**3.13**	**3.13**	**6.25**	**3.13**
76	12.50	**12.50**	12.50	12.50	**12.50**	12.50	12.50	**12.50**	12.50
A1	**12.50**	**12.50**	**12.50**	25.00	**25.00**	25.00	**6.25**	**12.50**	**6.25**
A2	**25.00**	**12.50**	**20.83**	**25.00**	**25.00**	**25.00**	**12.50**	**25.00**	**16.67**
A3	**12.50**	**12.50**	**12.50**	**12.50**	**12.50**	**12.50**	**12.50**	**12.50**	**12.50**
A4	**12.50**	**12.50**	**12.50**	**12.50**	**12.50**	**12.50**	**12.50**	**12.50**	**12.50**
M	6.25	**6.25**	6.25	6.25	**6.25**	6.25	6.25	**6.25**	6.25
*p* value^b^	< 0.001	< 0.001	< 0.001	< 0.001	< 0.001	< 0.001	< 0.001	< 0.001	< 0.001
stdev^c^	0.00–6.59			0.00–11.02			0.00–12.67		

^a^MIC values of combinations that are lower than the MIC of that honey alone are denoted in bold.

^b^ *p* values calculated using the t‐test, where *p* < 0.05 is considered significant.

^c^Standard deviations represented as a range.

The significant increase in the anti‐*H. pylori* activity seen when honey was combined with the *Lactobacillus*/*Lacticaseibacillus* species indicates that these species are inhibiting *H. pylori* growth. This was also seen when the *Lactobacillus*/*Lacticaseibacillus* species were investigated against *H. pylori* alone. The greatest increase in antimicrobial activity was seen in combinations with *L. casei*, where 82.50% of honey samples combined with *L. casei* had better activity than the respective honey alone against at least one strain. Up to a fourfold increase in activity was noted for four honey samples when combined with *L. casei*. These results are indicative of positive interactions between the investigated South African honey and the *Lactobacillus*/*Lacticaseibacillus* gut microbiome species.

*Lactobacillus*/*Lacticaseibacillus* species are noted to inhibit the growth of *H. pylori* by producing antimicrobials including lactic acid and H_2_O_2_. Lactic acid was found to inhibit urease, whereas H_2_O_2_ was noted to damage bacterial cell walls and membranes [42]. Several studies have investigated the anti‐*H. pylori* activity of probiotics, including *Lactobacillus*/*Lacticaseibacillus* species, combined with standard therapy, and found that the combinations improved *H. pylori* eradication and reduced side effects [42–44]. These results were, however, determined to be strain‐specific [42], which could account for the greater anti‐*H. pylori* activity noted for combinations with *L. casei* in this study.

Although honey combined with *Lactobacillus*/*Lacticaseibacillus* species was not found to be previously studied against *H. pylori*, honey combined with *Lactiplantibacillus plantarum* (formerly known as *Lactobacillus plantarum*) was found to inhibit wound pathogens both *in vitro* and *in vivo*, as well as accelerate wound healing [45]. A microbiota of diverse lactic acid bacteria, including *Lactobacillus*/*Lacticaseibacillus*, was found abundantly in bee honey crops. These lactic acid bacteria act as symbionts for bees, protecting them against microbial infections [46]. These symbionts are found abundantly in honey, where they were shown to produce a variety of extracellular proteins and antimicrobials [47]. The increase in anti‐*H. pylori* activity observed from the honey–*Lactobacillus*/*Lacticaseibacillus* combinations investigated in this study could thus be attributed to the longstanding symbiotic relationship between honeybees, the honey produced, and *Lactobacillus*/*Lacticaseibacillus* species.

### 3.3. H_2_O_2_ Content

**Figure 2:**
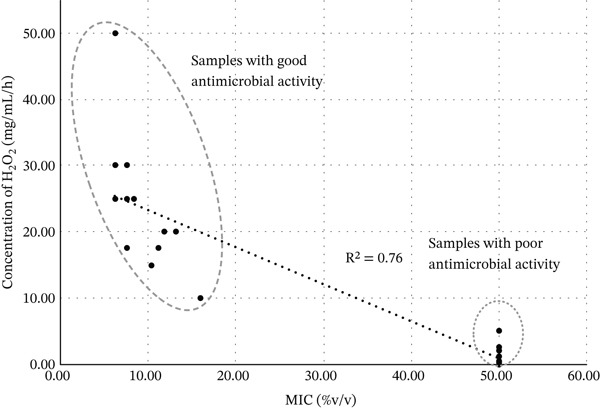
Correlation between the mean MIC values in % v/v of South African honey samples and the concentration of H_2_O_2_ in mg/mL/h.

The H_2_O_2_ concentrations were determined in selected honey samples (Table 4), with honey from blue gum, citrus, plum, and coriander floral sources showing the highest concentrations of H_2_O_2_. The R^2^ value calculated indicated a strong positive correlation between the antimicrobial activity against *H. pylori* and H_2_O_2_ concentration of tested honey samples (R^2^ = 0.76) (Figure 2). Honey samples with MIC values of ≥ 50.00% v/v held H_2_O_2_ concentrations between 0.00 and 5.00 mg/mL/h, whereas H_2_O_2_ concentrations of samples with noteworthy MIC values ranged from 10.00 to 50.00 mg/mL/h. No detectable H_2_O_2_ concentration was recorded for the Manuka honey sample.

**Table 4:** The hydrogen peroxide (H_2_O_2_) content in mg/mL/h of selected honey samples.

Honey sample	H_2_O_2_ (mg/mL/h)
**3** ^**a**^	**25.00** ^**a**^
**4**	**10.00**
**7**	**20.00**
**8**	**20.00**
**13**	**17.50**
15	0.00
**21**	**25.00**
22	0.00
**26**	**25.00**
**28**	**30.00**
29	5.00
34	0.00
**35**	**30.00**
36	2.00
37	0.50
**43**	**50.00**
52	0.25
54	1.25
**59**	**25.00**
**61**	**17.50**
62	2.50
**63**	**15.00**
**64**	**25.00**
67	2.50
**70**	**17.50**
M	0.00

^a^Samples with noteworthy MIC values denoted in bold.

Significant correlations between H_2_O_2_ concentrations and MICs have been previously reported [18]. A H_2_O_2_ concentration of 1.25 mg/mL/h was observed for the South African honey sample of *Eucalyptus* floral source in this study, while a maximum H_2_O_2_ concentration of 0.93 ± 0.12 mM was recorded for Australian honey of *Eucalyptus* floral source [19]. No detectable H_2_O_2_ was recorded for the South African orange blossom honey sample in this study. Similarly, low concentrations (0.01 ± 0.00 to 0.05 ± 0.01 mM) were recorded for orange blossom honey samples from Crete, Greece [48]. A study investigating Nigerian honey found that honey diluted with water was more effective against *H. pylori* compared with the undiluted sample [41]. This activity could be attributed to H_2_O_2_, as H_2_O_2_ is produced when honey is diluted with water [15, 16].

While other compounds and physicochemical factors of honey can contribute to antimicrobial activity, only two honey compounds, H_2_O_2_ and methylglyoxal, are classified as true antibacterials [18]. The Manuka honey sample investigated in this study had no detectable H_2_O_2_. Manuka honey has been noted to contain little to no detectable H_2_O_2_, with antimicrobial activity attributed to the high levels of methylglyoxal present [17, 49]. The anti‐*H. pylori* activity of South African honey observed in this study could thus be attributed to the H_2_O_2_ present in the samples upon dilution.

A wide variation was observed between the H_2_O_2_ concentrations recorded for the South African honey samples. H_2_O_2_ content has previously been noted to vary between honey samples, even when derived from the same floral source [19]. Factors affecting H_2_O_2_ accumulation include the age, storage, and processing of the honey, as glucose oxidase is sensitive to heat and light, as well as bee diet and health [19]. The presence of other compounds, such as catalase, metalloenzymes, and ascorbic acid, was found to reduce H_2_O_2_ accumulation in honey. Variation in H_2_O_2_ concentrations could also be the result of varying amounts of glucose oxidase present in the honey samples, as glucose oxidase is produced by bees during nectar collection and is thus dependent on bee health [18].

## 4. Conclusion

This study presents for the first time the anti‐*H. pylori* activity of a comprehensive range of South African honey samples. Varying antimicrobial activity against *H. pylori* was observed, with a third of the honey variants showing activity comparable with that of Manuka honey. The best anti‐*H. pylori* activity was demonstrated by honey produced using fynbos as the floral source. This flora is indigenous to South Africa. Honey sourced from citrus and coriander floral sources was found to possess high anti‐*H. pylori* activity as well. When the honey samples were combined with gut microbiome species, the activity against *H. pylori* was greatly increased, with combinations of honey and *L. casei* showing the greatest increase in antimicrobial activity. The correlation between the anti‐*H. pylori* activity of South African honey and the H_2_O_2_ concentrations present was investigated for the first time, with results indicating that the anti‐*H. pylori* activity of South African honey can be attributed to its H_2_O_2_ content.

While the results of this study demonstrate the antimicrobial effect of South African honey, investigation into the *in vivo* effects is required to determine the potential of South African honey for treating *H. pylori* infections and related ailments such as inflammation and gastric ulcers. Furthermore, investigation into the chemical composition of different honey samples could aid in the standardization of such natural products and provide insight into structure: anti‐*H. pylori* activity.

The results of this study present the unique honey of South Africa as an effective antimicrobial agent against *H. pylori*, not only by inhibiting *H. pylori* growth independently but also by enhancing this inhibition through interaction with gut microbiome species.

## Funding

The laboratory component of this study was supported by the National Research Foundation (NRF) of South Africa (Thuthuka Fund, Grant Number: 129672). The NRF is also acknowledged for postgraduate funding (MND200622534983).

## Ethics Statement

An ethical waiver was obtained from the University of the Witwatersrand Human Research Ethics Committee (W‐CBP‐210906‐01).

## Conflicts of Interest

The authors declare no conflicts of interest.

## Supporting information


**Supporting Information** Additional supporting information can be found online in the Supporting Information section. Table S1: Description of the southern African honey samples collected from beekeepers in conjunction with SABIO.

## Data Availability

The data that support the findings of this study are available from the corresponding author upon reasonable request.
