# Supplementary material for: South African Honey: Anti‐Helicobacter pylori Activity and Combined Effect With the Gut Microbiome
Source: Int J Food Sci. 2026 Jun 29;2026:9085243. doi: 10.1155/ijfo/9085243 (PMC13315830; doi:10.1155/ijfo/9085243)
Supplement: Supplementary file 1 — Supporting Information Additional supporting information can be found online in the Supporting Information section. Table S1: Description of the southern African honey samples collected from beekeepers in conjunction with SABIO. [file IJFO-2026-9085243-s001.docx]

**SUPPLEMENTARY DATA**

**TABLE S1:** Description of the Southern African honey samples collected from beekeepers in conjunction with SABIO.

| **Honey sample** | **Province** | **Specific area** | **Perceived floral source** | **Harvest date** |
| --- | --- | --- | --- | --- |
| 1 | Gauteng | Randburg | Unknown | Unknown |
| 2 | Gauteng | Sandton, IDC Grayston Drive | Unknown | 28 Jan 2022 |
| 3 | Gauteng | Sandton, Paulshof | Unknown | 13 Jan 2022 |
| 4 | Gauteng | Sandton, Riverclub | Unknown | 30 Jan /2022 |
| 5 | Gauteng | Sandton, Woodlands Eco Park | Unknown | Jan 2022 |
| 6 | Gauteng | SANS^a^ | Unknown | Unknown |
| 7 | Gauteng | SANS | Cannabis infused | Unknown |
| 8 | Gauteng | SANS | Bluegum | 24 May 2022 |
| 9 | Gauteng | SANS | Boekenhout | 24 May 2022 |
| 10 | Gauteng | SANS | Aloe | 24 May 2022 |
| 11 | Gauteng | SANS | Dombeya | 24 May 2022 |
| 12 | Gauteng | SANS | Light wild flora | 24 May 2022 |
| 13 | Gauteng | SANS | Wild flora | 24 May 2022 |
| 14 | Gauteng | SANS | Sunflower | 24 May 2022 |
| 15 | Gauteng | SANS | Orange blossom | 24 May 2022 |
| 16 | Gauteng | SANS | Summer multiflora | 24 May 2022 |
| 17 | Gauteng | SANS | Sideroxylon | 01 Jun 2022 |
| 18 | Gauteng | SANS | Buffalo thorn | 24 Mar 2022 |
| 19 | Gauteng | SANS | Macadamia | 24 May 2022 |
| 20 | Gauteng | SANS | Spring multiflora | 01 Jun 2022 |
| 21 | Gauteng | SANS | Buchu | 24 May 2022 |
| 22 | Kwa-Zulu Natal | Ladysmith | Carrot | Sep 2021 |
| 23 | Kwa-Zulu Natal | Midlands | Saligna gum | Aug 2021 |
| 24 | Kwa-Zulu Natal | Pietermaritzburg | Unknown | Dec 2021 |
| 25 | Limpopo | Amandelbult, Apiary 1 | Unknown | 01 Feb 2022 |
| 26 | Limpopo | Amandelbult, Apiary 2 | Unknown | 07 Feb 2022 |
| 27 | Limpopo | SANS | Unknown | Dec 2021 |
| 28 | Limpopo | SANS | Unknown | 13 Feb 2022 |
| 29 | Mpumalanga | Barberton | Macadamia | Nov 2021 |
| 30 | Mpumalanga | Kiepersol | Saligna gum | Nov 2021 |
| 31 | Mpumalanga | Malelane | Litchi | Nov 2021 |
| 32 | Western Cape | Barrydale | Navel oranges | Feb 2022 |
| 33 | Western Cape | Belvedere | Fynbos | Dec 2021 |
| 34 | Western Cape | Bonnievale | Prunes | Oct 2021 |
| 35 | Western Cape | Brenton-on-sea | Fynbos | Sep 2021 |
| 36 | Western Cape | Brenton-on-sea | Fynbos | Dec 2021 |
| 37 | Western Cape | Ceres, Bo-Swaarmoed | Ceres renosterveld | Feb 2022 |
| 38 | Western Cape | De Hoop | Coastal fynbos | Sep 2021 |
| 39 | Western Cape | De Hoop | Coastal fynbos | Feb 2022 |
| 40 | Western Cape | Gouritsmond | Coastal fynbos | Sep 2021 |
| 41 | Western Cape | Grabouw | Canola/apple | Nov 2021 |
| 42 | Western Cape | Grabouw | Apple | Nov 2021 |
| 43 | Western Cape | Heidelburg | Coriander | Feb 2022 |
| 44 | Western Cape | Heidelburg | Canola | Aug 2021 |
| 45 | Western Cape | Klaasvoogds | Naartjie | Nov 2021 |
| 46 | Western Cape | Knysna | Unknown | Dec 2021 |
| 47 | Western Cape | Knysna, Pezula | Unknown | Dec 2021 |
| 48 | Western Cape | Montagu | Vygie | Nov 2021 |
| 49 | Western Cape | Muldersvlei | Echium | Oct 2021 |
| 50 | Western Cape | Napier | Fynbos/Eucalyptus | Sep 2021 |
| 51 | Western Cape | Oudtshoorn | Aloe manor | Dec 2020 |
| 52 | Western Cape | Penhill | Mixed peri-urban | Nov 2021 |
| 53 | Western Cape | Plettenburg, Wittedrif | Natural bush | Dec 2021 |
| 54 | Western Cape | Robertson | Eucalyptus | Jun 2021 |
| 55 | Western Cape | SANS | Unknown | Unknown |
| 56 | Western Cape | SANS | Unknown | Unknown |
| 57 | Western Cape | SANS | Coriander | Nov 2021 |
| 58 | Western Cape | SANS | Almond | Nov 2021 |
| 59 | Western Cape | SANS | Citrus/plum | Nov 2021 |
| 60 | Western Cape | SANS | Apple | Nov 2021 |
| 61 | Western Cape | SANS | Mountain fynbos | Nov 2021 |
| 62 | Western Cape | SANS | Coastal fynbos | Nov 2021 |
| 63 | Western Cape | SANS | Grape | Nov 2021 |
| 64 | Western Cape | SANS | Plum | Nov 2021 |
| 65 | Western Cape | Sedgefield | Dune fynbos | Sep 2021 |
| 66 | Western Cape | Simondium | Echium | Nov 2021 |
| 67 | Western Cape | Stanford | Overberg fynbos | Nov 2021 |
| 68 | Western Cape | Stanford | Mixed urban forage | Nov 2021 |
| 69 | Western Cape | Swellendam | Canola | Aug 2021 |
| 70 | Western Cape | Swellendam | River red gum / African basil | Feb 2022 |
| 71 | Western Cape | Tesselaarsdal | Canola / fynbos | Sep 2021 |
| 72 | Western Cape | Touwsrivier | Karoo veld | Feb 2022 |
| 73 | Western Cape | Wellington | Almonds | Sep 2021 |
| 74 | Western Cape | West Coast | West Coast wild flora | 24 May 2022 |
| 75 | Western Cape | Wilderness | Unknown | Nov 2021 |
| 76 | Western Cape | Yzerfontein | West Coast fynbos | Nov 2021 |
| A1 | - | Malawi | Indigenous flora | Unknown |
| A2 | - | Malawi | Red forest gum | Unknown |
| A3 | - | Malawi | Indigenous flora | Unknown |
| A4 | - | Mozambique | Macadamia | Unknown |
| M | - | New Zealand | Manuka (MGO 67+) | Unknown |

^a^ Specific area not specified.
